# Electroacupuncture Improves Cardiac Function via Inhibiting Sympathetic Remodeling Mediated by Promoting Macrophage M2 Polarization in Myocardial Infarction Mice

**DOI:** 10.1155/2024/8237681

**Published:** 2024-06-30

**Authors:** Rou Peng, Junjing Shi, Minjiao Jiang, Danying Qian, Yuhang Yan, Hua Bai, Meiling Yu, Xin Cao, Shuping Fu, Shengfeng Lu

**Affiliations:** ^1^ Key Laboratory of Acupuncture and Medicine Research of Ministry of Education Nanjing University of Chinese Medicine, Nanjing 210023, China; ^2^ The Second People's Hospital of Qidong, South Ring Road No. 229, Lvsigang Town, Qidong, Jiangsu Province 226200, China; ^3^ Acupuncture and Chronobiology Key Laboratory of Sichuan Province Acupuncture and Tuina School/Third Teaching Hospital Chengdu University of Traditional Chinese Medicine, Chengdu 610075, China; ^4^ School of Elderly Care Services and Management Nanjing University of Chinese Medicine, Nanjing 210023, China

## Abstract

Electroacupuncture (EA) at the Neiguan acupoint (PC6) has shown significant cardioprotective effects. Sympathetic nerves play an important role in maintaining cardiac function after myocardial infarction (MI). Previous studies have found that EA treatment may improve cardiac function by modulating sympathetic remodeling after MI. However, the mechanism in how EA affects sympathetic remodeling and improves cardiac function remains unclear. The aim of this study is to investigate the cardioprotective mechanism of EA after myocardial ischemic injury by improving sympathetic remodeling and promoting macrophage M2 polarization. We established a mouse model of MI by occluding coronary arteries in male C57/BL6 mice. EA treatment was performed at the PC6 with current intensity (1 mA) and frequency (2/15 Hz). Cardiac function was evaluated using echocardiography. Heart rate variability in mice was assessed via standard electrocardiography. Myocardial fibrosis was evaluated by Sirius red staining. Levels of inflammatory factors were assessed using RT-qPCR. Sympathetic nerve remodeling was assessed through ELISA, western blotting, immunohistochemistry, and immunofluorescence staining. Macrophage polarization was evaluated using flow cytometry. Our results indicated that cardiac systolic function improved significantly after EA treatment, with an increase in fractional shortening and ejection fraction. Myocardial fibrosis was significantly mitigated in the EA group. The sympathetic nerve marker tyrosine hydroxylase and the nerve sprouting marker growth-associated Protein 43 were significantly reduced in the EA group, indicating that sympathetic remodeling was significantly reduced. EA treatment also promoted macrophage M2 polarization, reduced levels of inflammatory factors TNF-*α*, IL-1*β*, and IL-6, and decreased macrophage-associated nerve growth factor in myocardial tissue. To sum up, our results suggest that EA at PC6 attenuates sympathetic remodeling after MI to promote macrophage M2 polarization and improve cardiac function.

## 1. Introduction

Myocardial infarction (MI) is a significant global health concern [1] and can progress to malignant ventricular arrhythmias (VAs) and heart failure (HF) [2, 3]. Considerable evidence has been shown that post-MI complications have a correlation between cardiac sympathetic nerve sprouting and cardiac electrical remodeling initiated by infarction [4]. In the post-MI phase, autonomic denervation and neural remodeling occur in the peri-infarct region. This remodeling process involves the excessive growth of sympathetic nerves compared to parasympathetic nerves, contributing to sympathetic overinnervation [5]. Excessive sympathetic reinnervation can disrupt electrical and myocardial remodeling, leading to instability and functional decline in the heart [6, 7, 8]. Inhibiting sympathetic regeneration after MI may be an effective strategy to prevent malignant arrhythmias and improve cardiac function. Further research needs to explore innovative approaches for modulating sympathetic overinnervation, restoring autonomic balance, and improving patient outcomes by reducing the burden of post-MI complications.

Sympathetic nerve remodeling is a complex pathological process after MI. Recent research has indicated a close association between sympathetic remodeling and inflammation following MI [9]. Increased inflammation, accompanied by activation of the sympathetic nervous system, promotes the remodeling of the stellate ganglia, thereby facilitates ischemia-induced VAs [10]. More recent studies have emphasized the interaction between macrophages and sympathetic nerves, as neuro-associated macrophages can directly interact with catecholamines released by the sympathetic nervous system. This interaction activates *β*-adrenergic receptors expressed on macrophages, inducing sympathetic nerve remodeling [11]. Conversely, macrophages have the capability to produce catecholamines, allowing them to modulate inflammatory damage [12]. Nerve growth factor (NGF) has been shown to play a crucial role in the growth, survival, differentiation, and configuration of sympathetic nerves [13]. Upregulation of NGF is a major factor in promoting sympathetic nerve sprouting and remodeling after MI [14, 15]. Macrophages are such vital places where NGF synthesizes and mediates the inflammatory response and cardiac conduction after MI. Synthesized NGF from infiltrating macrophages contributes to excessive sympathetic innervation after MI [16]. Studies have shown that depleting macrophages using clodronate can reduce NGF levels in myocardial tissue, preventing excessive sympathetic innervation and improving myocardial electrical remodeling in the peri-infarct region [11, 16]. Furthermore, promoting macrophage M2 polarization has been found to significantly decrease NGF levels [17, 18, 19, 20]. Currently, macrophages are generally categorized as pro-inflammatory M1 or anti-inflammatory M2 macrophages. M1 macrophages playing a dominant role in cell infiltration, debris clearance, and the production of high levels of oxidative metabolites and pro-inflammatory cytokines, while M2 macrophages coordinate the subsequent phases of inflammation resolution, healing, and tissue regeneration by inhibiting detrimental immune responses [21, 22]. Therefore, promoting macrophage M2 polarization may be beneficial in influencing sympathetic nerve remodeling following MI.

Acupuncture has become a nonpharmacological therapy for the prevention and treatment of cardiovascular diseases, including angina, palpitations, and HF [23, 24, 25]. Moreover, it has been shown that acupuncture at Neiguan (PC6) acupoint can inhibit premature ventricular complexes after MI by reducing inflammation and fibrosis [26]. Electroacupuncture (EA), a technique that involves stimulating acupuncture points with low-voltage electrical currents, has gained popularity as an alternative form of acupuncture [27]. Among the various acupuncture points, the PC6 on the Pericardium Meridian of Hand Jueyin is widely used for the treatment of cardiovascular conditions [28, 29]. Furthermore, previous researches have demonstrated that EA can effectively reduce myocardial damage and limit the extent of MI [30, 31]. Our previous studies have provided further insights into the potential mechanisms not only behind EA pretreatment but also behind EA treatment at PC6 [32, 33, 34]. We have found that it can inhibit the infiltration of immune cells such as macrophages and neutrophils, thereby alleviating inflammatory reactions, improving cardiac function, and regulating the cardiac autonomic imbalance followed by MI. However, how the EA affects sympathetic remodeling and thus improves cardiac function remains unclear, and the exact underlying mechanisms remain to be elucidated.

Therefore, based on the clear understanding that EA at PC6 for 28 days (three times per week) has effects on cardiac protection, including reducing myocardial fibrosis, regulating heart rate variability (HRV), and reducing macrophage infiltration. This study aims to further investigate the dynamic changes in macrophage polarization states on Day 1, Day 3, and Day 7 after ischemic injury. Additionally, this study will focus on exploring the cardioprotective effects on myocardial fibrosis, HRV, substances related to sympathetic nerve remodeling, macrophage infiltration, and polarization, as well as inflammation levels in 7 days after MI with or without EA treatment at PC6.

## 2. Materials and Methods

### 2.1. Experimental Animals and Grouping

**Figure 1:**
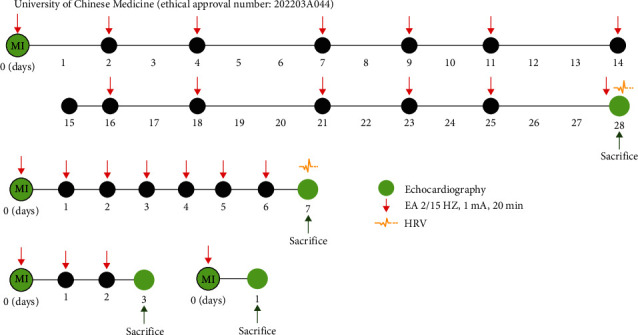
Experimental protocol.

Male C57BL/6J SPF mice (6–8 weeks old, weighing 20–22 g, *n* = 93) were obtained from SPF (Beijing, China) Biotechnology Co., Ltd. (NO. SCXK 2019-0010), housed with ad libitum access to water and food and were acclimated for 1 week under controlled conditions of a 12-hr light/12-hr dark cycle, an ambient temperature of (25 ± 2)°C, and a relative humidity of (45 ± 5)%. They were randomly divided into three groups, namely the sham-operated (Sham), model (MI), and EA groups, using a random number table method. Additionally, for the experiments at different time-points (1 day, 3 days, and 7 days), mice were grouped accordingly. The experimental protocol is shown in Figure 1. All experimental procedures were conducted in accordance with the institutional animal care guidelines and were approved by the Animal Ethics Committee of Nanjing University of Chinese Medicine (ethical approval number: 202203A044).

### 2.2. Induction of MI in Mice

MI models were established by ligation of the left anterior descending (LAD) coronary artery. The surgical procedures were performed as previously described [32, 35]. Mice were rapidly anesthetized with 5% isoflurane (RWD, China), and a thermostatic heating pad was used to maintain a constant temperature of 37°C, followed by 2% isoflurane to maintain anesthesia. The left anterior thoracic skin of the mice was then incised, the muscle was bluntly dissected, the 4th intercostal space was penetrated, and the LAD coronary artery branch was permanently ligated with a 6/0 monofilament suture, and the muscle layer and skin incisions were then closed with 3/0 monofilament sutures (Shanghai Pudong Jinhuan Medical Instrument Co., Ltd., China) to induce myocardial ischemia. The Sham group underwent the same procedure, except that the LAD branch was ligated. The Lead II electrocardiogram was monitored by the use of a computerized Power Lab system (AD Instruments, Australia) before and after the operation. The ST segment of the electrocardiogram was elevated to the myocardial ischemic indication.

### 2.3. EA Intervention

The EA group was treated with an EA intervention at the bilateral PC6 acupoint half an hour after the MI procedure. This was followed by one session per day or three sessions per week. The needle was folded into an inverted “L” shape and inserted into the muscle layer between the bilateral PC6 palmar tendon and the ulnar carpal flexor at a depth of 3 mm. A Han's acupoint nerve stimulator (HANS-200A, China) was attached to deliver current at an EA frequency of 2/15 Hz and an intensity of 1 mA for 20 min.

### 2.4. Echocardiography

Mice underwent transthoracic echocardiography under 2% isoflurane for M-mode echocardiography using a high-resolution imaging system equipped (Esaote, Italy) with an SL3116 transducer (10–22 MHz), where we measured heart rate (maintaining at about 450–550 bpm during the examination), left ventricular end-diastolic diameter (LVEDD) and left ventricular end-systolic diameter (LVESD). Left ventricular ejection fraction (LVEF) and left ventricular fractional shortening (LVFS) were calculated from LVEDD and LVESD. The following equation was used: LVEF% = (LVEDD^3^−LVESD^3^)/ LVEDD^3^ × 100%; LVFS% = (LVEDD−LVESD)/ LVEDD × 100% [36].

### 2.5. HRV Analysis

Mice were maintained on 2% isoflurane after rapid induction of anesthesia with 5% isoflurane using an inhalation anesthesia machine, and body surface ECGs were recorded for 20 min under anesthesia using a computerized Power Lab system (AD Instruments, Australia). The more stable 5-min segments of the ECG were selected for HRV analysis (Lab Chart 8.0, AD Instruments). Power spectrum variables included low frequency (LF, 0.20–0.75 Hz), high frequency (HF, 0.75–2.5 Hz), and LF/HF ratio and were calculated as previously described [37, 38].

### 2.6. Sirius Red Staining

At the end of the experiments, the mice were perfused with PBS or Fixative. Heart tissue below the ligature line was rapidly fixed in 4% paraformaldehyde and embedded in paraffin. Paraffin blocks of heart tissue were sectioned and placed sequentially in glass vials containing different dewaxing solutions: first in two vials of xylene solution for 20 min each, then in two vials of anhydrous ethanol solution for 5 min each, then in 75% ethanol solution for 5 min, and finally in distilled water for 5 min, the sections were stained in Sirius red staining solution for 8 min, washed with distilled water and then cleared in anhydrous ethanol solution, n-butanol solution and xylene solution. The sections were air-dried and embedded in neutral resin. Images were acquired using a microscope (Nikon, Japan) with a fully automated quantitative pathology imaging system (PerkinElmer, USA), and all quantitative analyses were performed using commercial software (Image J, NIH, Bethesda, MD, USA).

### 2.7. Histological Staining

Heart tissue below the ligature line was removed and immersion-fixed in 4% paraformaldehyde for histological study. The heart is innervated by sympathetic, parasympathetic, and sensory fibers, of which sympathetic nerves are usually stained by TH [18, 39]. Paraffin blocks of heart tissue were processed and cut into 4-*μ*m sections for immunohistochemistry for TH (1 : 2,000, CST, USA), and together with GAP43 [19, 32] (1 : 200, Immunoway, USA), these two antibodies were used to assess cardiac sympathetic innervation. In addition, immunofluorescence staining for F4/80 (1 : 200, CST, USA) and NGF (1 : 300, Abcam, UK) was used to assess the contribution of macrophages to NGF. Cell nuclei were stained with 4′, 6-diamidino-2-phenylindole (DAPI). Images were acquired using a microscope (Nikon, Japan) and a fully automated quantitative pathology imaging system (PerkinElmer, USA), and quantitative analysis was performed using commercial software (Image J, NIH, USA).

### 2.8. Flow Cytometry

After execution of each group of mice, the myocardial tissue below the ligature line was washed with ice-cold PBS, the left ventricular tissue was removed and cut it up as much as possible, and then placed in a 15 ml centrifuge tube with 5 ml cardiac digestion solution (RPMI 1640 medium solution with type I collagenase: 1.6 mg/ml and deoxyribonuclease I: 0.2 mg/ml, Sigma Chemical Co., USA) and mixed intermittently at 37°C. After digestion for 1 hr, the tissue was filtered through a 40 *μ*m nylon mesh cell filter, centrifuged at 1,500 rpm/min at 4°C and the supernatant discarded. The tissue was then resuspended with 200 *µ*l erythrocyte lysate, protected from light for 5–10 min at 4°C, centrifuged, the supernatant discarded, and resuspended with 200 ml ice-cold PBS. Surface Fc receptors were blocked with anti-CD16/32 antibody (eBioscience, USA) for 5 min at 4°C, and cells were then stained with specific antibodies. Cells were stained with antimouse FITC-conjugated F4/80 antibody (eBioscience, USA), antimouse APC-conjugated CD11b antibody (eBioscience, USA), and antimouse PE-conjugated CD206 antibody (eBioscience, USA). The data were analyzed utilizing a CytoFLEX instrument (Beckman Coulter, Inc., USA) and the CytExpert software. Gating strategies were employed to detect different subsets of infiltrating immune cells. Initially, immune cells were gated on a forward scatter (FSC)/side scatter (SSC) plot. Further gating was conducted to identify macrophages (F4/80^+^CD11b^+^). Based on the expression levels of CD206, the F4/80^+^CD11b^+^ cells were then subdivided into M1 (F4/80^+^CD11b^+^CD206^low^) and M2 (F4/80^+^ CD11b^+^CD206^high^) macrophages [35].

### 2.9. ELISA

Blood was collected from each mouse at the time of sacrifice, stored in anticoagulant-free tubes, and allowed to stand at room temperature for 30 min. The serum was then extracted by centrifugation at 3,000 rpm for 15 min; the supernatant was separated from the myocardial tissue by centrifugation at 3,000 rpm for 15 min. Norepinephrine (NE) concentrations in serum and tissue supernatants were determined using a mouse NE ELISA kit (Jinyibai, China) according to the manufacturer's instructions.

### 2.10. RT-qPCR

Total mRNA was isolated from heart tissue below the ligation line using Trizol (Vazyme, China) and Fast Pure Cell/Tissue Total RNA Isolation Kit (RC101-01, Vazyme, China), and the concentration of isolated RNA was determined using a spectrophotometer (Thermo Fisher Scientific, USA), followed by Strand cDNA Synthesis SuperMix (YEASEN, China) for cDNA preparation. The cDNA was prepared on an Applied Biosystems ViiA™ 7 Real-Time PCR system (Applied Biosystems) using SYBR Green reagent (YEASEN, China) to assess mRNA levels. The relative expression of mRNAs was calculated using *∆∆*^Ct^ according to standard methods. The primer sequences used in this study are listed in Table 1.

### 2.11. Western Blotting

Mouse heart tissue below the ligation line was crushed in liquid nitrogen, sufficiently lysed by the addition of RIPA lysis solution (Beyotime Biotechnology, China), and the supernatant was centrifuged to extract total protein. The protein concentration was measured using the BCA Protein Assay Kit (Beyotime Biotechnology, China), and then a loading buffer (CWBIO, China) was added and denatured at 100°C for 5 min. Initially, the sample proteins were added to each of the remaining wells, with 14 *μ*g of protein sampled in each well, analyzed by 10% SDS–PAGE, and then transferred to a PVDF membrane (Merck and Millipore, USA). The membranes were then blocked with 5% BSA, and after sealing, the PVDF membranes were immersed in specific primary antibodies (TH, 1 : 2,000, Proteintech, China; NGF, 1 : 1,000, Abcam, UK; GAPDH 1 : 10,000, CST, USA; *β*-tubulin, 1 : 1,000, Proteintech, China) overnight at 4°C in a shaker. The membranes were then washed with TBST solution (10 mM Tris-HCl, 150 mM NaCl, and 0.5% Tween-20) and incubated with antirabbit horseradish peroxidase secondary antibody (1 : 500; Solarbio, China) and visualized by ECL kit (Jiyi, China) in a chemiluminescence imaging system (VILBER BIO IMAGING, France). Bands were quantified using Image J software (NIH, USA).

### 2.12. Statistical Analysis

All data are presented as the mean ± SEM. Statistical analysis was performed using one-way analysis of variance (ANOVA) with Tukey's test using GraphPad Prism 9.0 (GraphPad Software, USA) and SPSS 27.0 software (IBM, USA). A value of *P* < 0.05 was considered to be statistically significant.

## 3. Results

### 3.1. EA Treatment Improved Cardiac Function and Reduced Cardiac Fibrosis at 28 Days after MI

**Figure 2:**
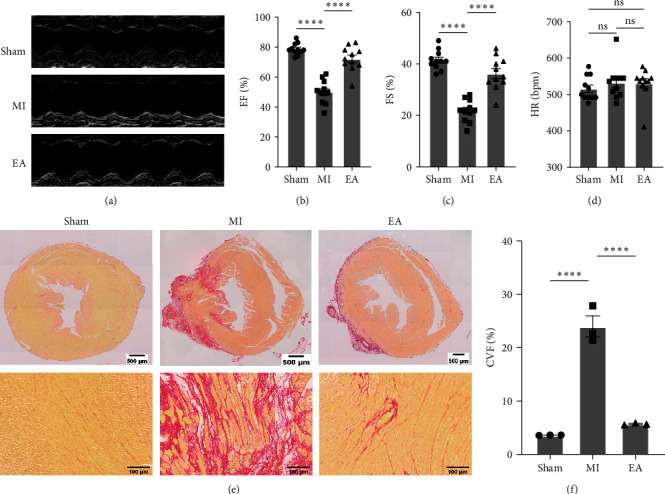
EA treatment improved post-MI cardiac function and reduced fibrosis. (a) Representative M-mode of echocardiographic images of mice at 28 days after MI (*n* = 11 per group) are shown. (b–d) Quantitative group data for EF (b), FS (c), and HR (d) are shown (*n* = 11 per group). (e) Representative images of Sirius red staining are shown at 28 days after MI (*n* = 3 per group). Scale bar =100 *μ*m. (f) Quantitative analysis of Sirius red staining in all groups (*n* = 3 per group). EF, ejection fraction; FS, fractional shortening; HR, heartbeats per minute. Data are shown as mean ± SEM. ns, no significance,  ^*∗∗∗∗*^*P* < 0.0001.

Example echocardiograms are shown in Figure 2(a). Echocardiography was used to evaluate the impact of EA treatment for 4 weeks (3 times per week) on post-MI cardiac function. Indicators of left ventricular systolic function, EF, and FS were significantly lower in the MI group compared to the Sham group, as shown in Figures 2(b) and 2(c). However, the reduction in EF and FS could be effectively reversed by EA treatment, with the heart rate maintained at the time of measurement, as illustrated in Figure 2(d). Ventricular Sirius red staining was employed to investigate the effects of EA treatment on cardiac fibrosis after MI. The results demonstrated a substantial reduction in ventricular fibrosis within the infarcted region of the MI group following EA treatment (Figure 2(e) and 2(f)). These findings suggest that EA treatment is a successful intervention to mitigate myocardial fibrosis and exerts a protective effect on heart function.

### 3.2. EA Treatment Suppressed Sympathetic Nerve Activity and Reduced Macrophage Infiltration at 28 Days after MI

**Figure 3:**
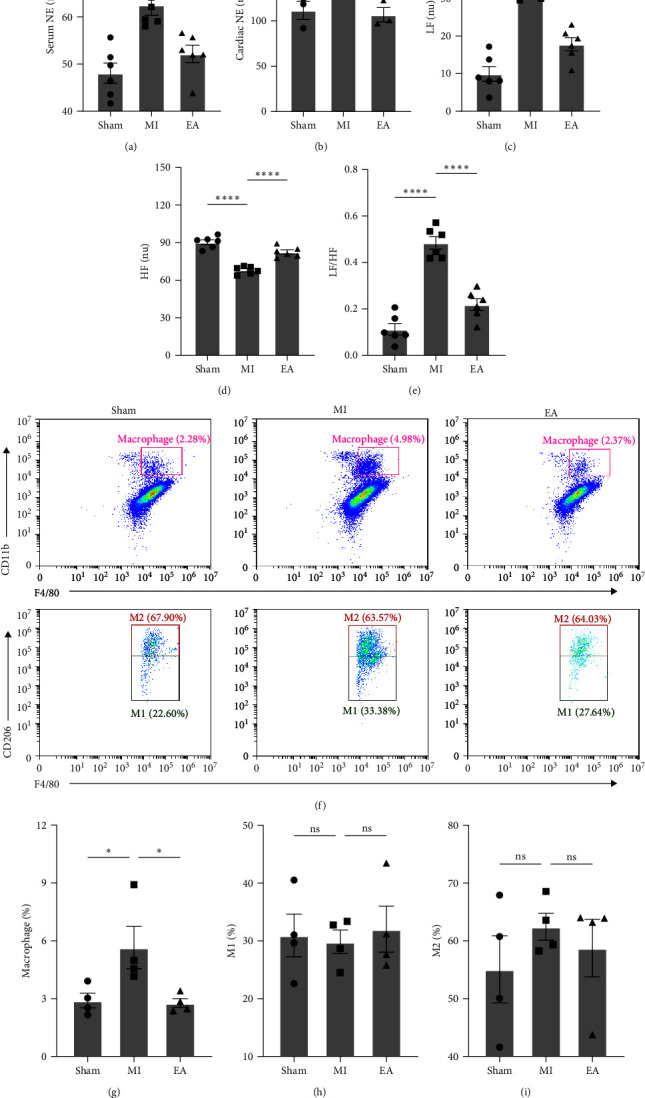
EA treatment inhibited post-MI systemic sympathetic activity and reduced macrophage infiltration: (a) serum NE levels at 28 days after MI (*n* = 6 per group); (b) heart tissue NE levels 28 days after MI (*n* = 3 per group); (c–e) spectral analysis of heart rate variability reflecting sympathetic activity: ratios of LF (c), HF (d), and LF/HF (e) (*n* = 6 per group); (f) Representative flow cytometric analysis of macrophage infiltration in the heart 28 days after MI (*n* = 4 per group). (g–i) Representative flow cytometric analysis of M1 and M2 macrophages in the heart at 28 days after MI (*n* = 4 per group). NE, noradrenaline; LF, low frequency (0.20–0.75 Hz); HF, high frequency (0.75–0.2 Hz); LF/HF, low-frequency/high-frequency ratio. Data are shown as mean ± SEM. ns, no significance,  ^*∗*^*P*  < 0.05,  ^*∗∗*^*P*  < 0.01,  ^*∗∗∗*^*P*  < 0.001,  ^*∗∗∗∗*^*P*  < 0.0001.

ELISA was employed to measure NE levels, confirming sympathetic activation. After 28-days of EA treatment following MI, we focused on the infarct zone and serum NE levels. Both the infarct zone and serum NE levels were elevated. However, EA treatment effectively slowed down the MI-induced increase in NE levels, as depicted in Figures 3(a) and 3(b). To assess cardiac autonomic function, HRV was examined (Figures 3(c), 3(d), and 3(e)). In comparison to the Sham group, MI resulted in an increase in LF and LF/HF, along with a decrease in HF, indicating sympathetic activation. However, EA treatment significantly decreased LF and LF/HF, while increasing HF. These findings suggest that EA treatment has the potential to improve cardiac autonomic balance followed MI. By reducing sympathetic activation and increasing parasympathetic activity, EA treatment may contribute to the restoration of cardiac autonomic function. We conducted an investigation to examine the impact of EA treatment on macrophages 28 days post-MI. The results revealed that EA treatment led to a significant reduction in the proportion of elevated macrophages after MI, as illustrated in Figure 3(f) and 3(g). However, it was observed that EA treatment did not have a significant effect on macrophage M2 polarization (Figure 3(f), 3(h), and 3(i)).

### 3.3. EA Treatment Improved Cardiac Function, Reduced Cardiac Fibrosis, and Suppressed Sympathetic Nerve Activity at 7 Days after MI

**Figure 4:**
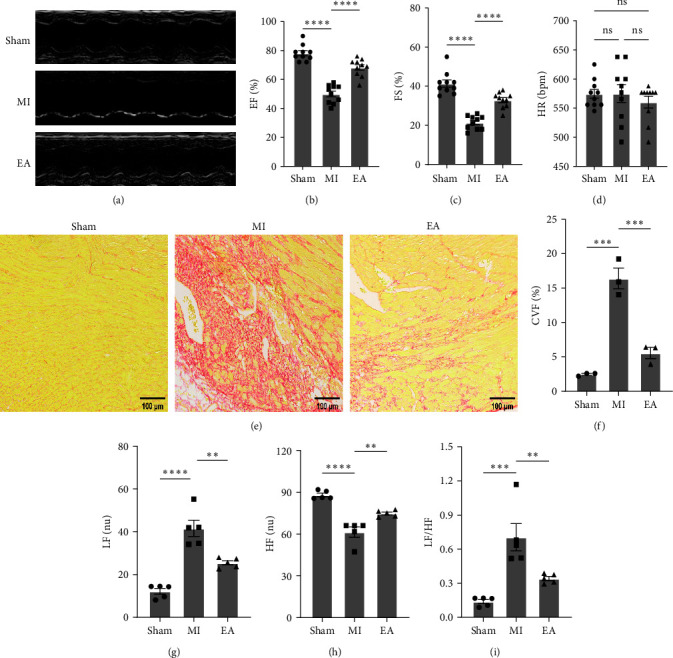
EA treatment improved post-MI cardiac function, reduced cardiac fibrosis and decreased sympathetic activation. (a) Representative M-mode echocardiographic images of mice at 7 days after MI are shown (*n* = 10 per group). (b–d) Quantitative groups data for EF (b), FS (c), and HR (d) are shown (*n* = 10 per group). (e) Representative images of Sirius red staining are shown at 7 days after MI (*n* = 3 per group). Scale bar = 100 *μ*m. (f) Quantitative analysis of Sirius red staining in all groups (*n* = 3 per group). (g–i) Spectral analysis of heart rate variability reflecting sympathetic activity: ratios of LF (g), HF (h) and LF/HF (i) (*n* = 5 per group). EF, ejection fraction; FS, fractional shortening; HR, heartbeats per minute. Data are shown as mean ± SEM. ns, no significance,  ^*∗∗*^*P*  < 0.01,  ^*∗∗∗*^*P*  < 0.001,  ^*∗∗∗∗*^*P*  < 0.0001.

Typical echocardiograms of the functional and structural effects on the heart during 7 days of EA treatment are shown in Figure 4(a). As shown in Figures 4(b) and 4(c), EF and FS were significantly higher in the EA group compared to the MI group, and these data were measured while maintaining a relatively stable heart rate (Figure 4(d)). Sirius red staining showed that EA treatment significantly reduced the degree of ventricular fibrosis in the MI group (Figures 4(e) and 4(f)). As described above, we analyzed HRV to assess cardiac autonomic function. LF and LE/HF were significantly lower and HF was significantly higher in the EA group compared with the MI group (Figures 4(g), 4(h), and 4(i)).

### 3.4. EA Treatment Reduced Macrophage Infiltration, Promoted Macrophage M2 Polarization, and Reduced Inflammatory Factor Infiltration at 7 Days after MI

**Figure 5:**
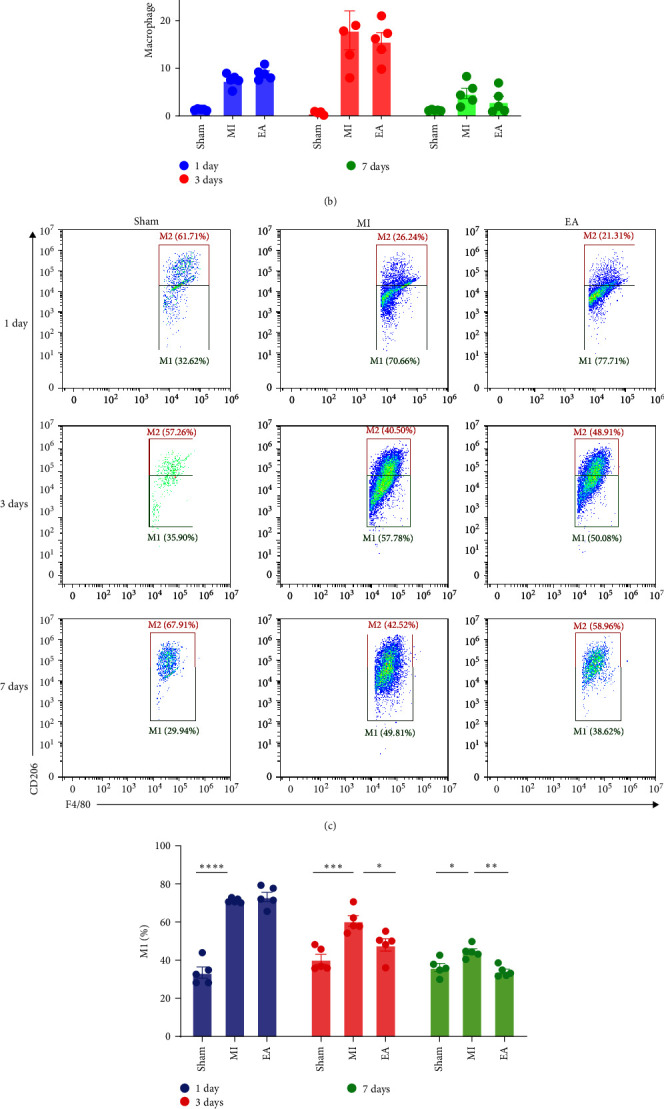
Effect of EA treatment on post-MI macrophage infiltration, macrophage polarization, and inflammatory factor: (a and b) representative flow cytometric analysis of macrophage infiltration in the heart at 1, 3, and 7 days after MI (*n* = 5 per group); (c–e) representative flow cytometric analysis of M1 and M2 macrophages in the heart at 1, 3 and 7 days after MI (*n* = 5 per group); (f–h) expression of inflammatory cytokines TNF-*α* (*n* = 5 per group), IL-1*β* (*n* = 5 per group) and IL-6 (*n* = 3 per group) in myocardium at 7 days after MI was assessed by RT-qPCR. Data are expressed as mean ± SEM. ns, no significance,  ^*∗*^*P*  < 0.05,  ^*∗∗*^*P*  < 0.01,  ^*∗∗∗*^*P*  < 0.001,  ^*∗∗∗∗*^*P*  < 0.0001.

We conducted a study to investigate the effect of EA treatment on macrophages in the heart tissue of mice in 28 days after MI. To observe the dynamic effect of EA treatment on macrophages, flow cytometry was performed to analyze macrophage recruitment and polarization at different time points according to the experimental protocol. The results, as shown in Figures 5(a) and 5(b), revealed that 7 days of EA treatment significantly reduced macrophage infiltration, when compared to 3 days of EA treatment after MI. Additionally, both 3-day and 7-day treatment of EA promoted M2 polarization in macrophages, while 1-day treatment of EA did not have a significant effect (Figures 5(c), 5(d), and 5(e)). Importantly, the ratio of 7-day EA treatment exhibited a distinct difference in reducing macrophage infiltration and promoting M2 polarization compared to the MI group. TNF-*α*, IL-1*β*, and IL-6 are considered crucial inflammatory cytokines involved in chronic inflammation during ventricular remodeling. The levels of these inflammatory factors were reduced in the EA group compared to the MI group (Figure 5(f), 5(e), 5(g), and 5(h)). These findings suggest that EA treatment promotes M2 polarization in macrophages, reduces inflammation levels, and modulates the inflammatory response.

### 3.5. EA Treatment Downregulated the Expression of GAP43 and TH at 28 Days after MI

**Figure 6:**
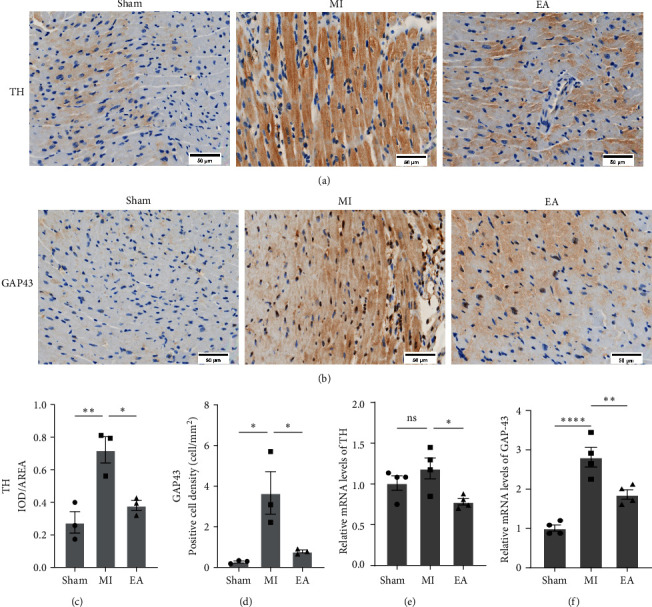
Effect of EA treatment on post-MI cardiac sympathetic nervous system remodeling in mice post-MI. (a, c) Representative TH immunohistochemical staining and analysis of heart tissues in 28 days after MI (*n* = 3 per group), scale bar = 50 *μ*m. (b, d) Representative GAP43 immunohistochemical staining and analysis of heart tissues at 28 days after MI (*n* = 3 per group). Scale bar = 50 *μ*m. (e) The relative mRNA expression of TH in heart (*n* = 4 per group). (f) The relative mRNA expression of GAP43 in the heart (*n* = 4 per group). Data are expressed as mean ± SEM. ns, no significance,  ^*∗*^*P*  < 0.05,  ^*∗∗*^*P*  < 0.01,  ^*∗∗∗∗*^*P*  < 0.0001.

In this study, immunohistochemistry was employed to investigate the effects of EA treatment on sympathetic nerve activity in the heart by examining sympathetic nerve remodeling after MI. The expression of the sympathetic marker TH and the nerve sprout marker GAP43 was analyzed. Figures 6(a) and 6(b) illustrate that at 28 days after MI, the MI group exhibited a significant increase in sympathetic nerve fibers and nerve sprouting in the area surrounding the MI compared to the Sham group. Conversely, the EA group demonstrated a significant decrease in sympathetic nerve activity. The relative quantitative evaluation of TH and GAP43 expression is presented in Figures 6(c) and 6(d). Furthermore, the mRNA expression of GAP43 displayed a similar trend (Figure 6(e)), whereas there was no significant change in TH mRNA expression in the MI group and Sham group (Figure 6(f)). This discrepancy may be attributed to the difference in expression timing between protein and mRNA levels. In conclusion, these findings suggest that MI induces sympathetic remodeling. However, treatment with EA effectively ameliorates these adverse reactions, thereby improving cardiac sympathetic remodeling.

### 3.6. EA Inhibited MI-Induced NGF Production and Downregulated the Expression of TH and NGF at 7 Days after MI

**Figure 7:**
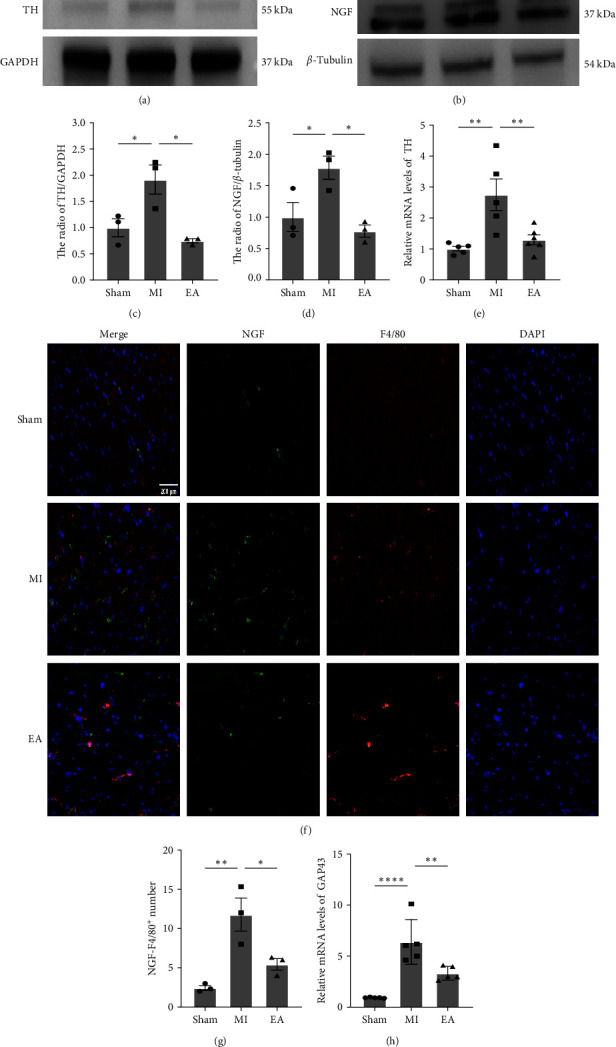
Effects of EA treatment on the MI-induced NGF production and the expression of GAP43 and TH post-MI. (a, c) Representative Western blots and quantification of NGF expression (*n* = 3 per group). (b, d) Representative Western blots and quantification of TH expression (*n* = 3 per group). (e) TH mRNA levels in cardiac tissue at 7 days (*n* = 5 per group). (f) Representative images of immunofluorescence staining for NGF/F480+ cell expression in the heart at 7 days (*n* = 3 per group). Positive staining for F4/80 is red and NGF is green. Cell nuclei were counterstained with DAPI (blue). Scale bar = 200 *μ*m.(g) Immunostaining quantification of staining for NGF/F4/80+ cells at 7 days after (*n* = 3 per group). (h) GAP43 mRNA levels in cardiac tissue 7 days after (*n* = 5 per group). Data are shown as mean ± SEM. ns, no significance,  ^*∗*^*P*  < 0.05,  ^*∗∗*^*P*  < 0.01,  ^*∗∗∗∗*^*P*  < 0.0001.

Considering sympathetic hyperactivity could reach to the peak on day 7 after MI [40], we re-examined sympathetic markers TH and NGF in each group of mice with Western blot analysis. Elevated TH and NGF were found in the MI group, but this upregulation was reversed after EA intervention (Figures 7(a), 7(b), 7(c), and 7(d)). RT-qPCR analysis of TH in transcript levels showed that EA treatment decreased upregulated TH expression in the MI group (Figure 7(e)). Immunofluorescence detection of NGF-positive signals in cardiac macrophages (NGF/F4/80^+^ cells) in the peri-infarct zone of each group of mice showed a significant increase in NGF/F4/80^+^ cells in the MI group compared to the Sham group, while NGF-positive expression was reduced in mice treated with EA (Figures 7(f) and 7(g)). Similarly, EA treatment effectively reduced the expression of expression of GAP43 (Figure 7(h)). These results indicate that EA treatment can effectively inhibit sympathetic remodeling induced by macrophages.

## 4. Discussion

In this study, we utilized a mouse model of ischemia MI to investigate the cardioprotective mechanism of EA treatment on sympathetic nerve remodeling and macrophage polarization. We monitored various indicators of mice hearts for 7 and 28 days to assess the potential protective effects of EA treatment from long-term and short-term aspects. Our findings revealed that EA treatment enhanced macrophage M2 polarization, reduced myocardial fibrosis followed by MI, decreased cardiac sympathetic nerve activity, regulated autonomic balance, prevented sympathetic remodeling after MI, improved cardiac contractile function, and exerted cardioprotective effects. These results provide scientific and experimental evidence supporting the ability of EA treatment to improve cardiac remodeling and prognosis after MI. This research has significant implications for the treatment options of cardiac patients and the promotion and application of the EA therapy.

MI can lead to necrosis of the myocardial cells due to prolonged local tissue ischemia and hypoxia. To repair the necrotic myocardium, the body induces fibroblasts to synthesize collagen and form scar tissue [41]. Myocardial fibrosis is a key determinant of myocardial heterogeneity and serves as the foundation for cardiac remodeling [42]. However, excessive collagen accumulation worsens cardiac function and contributes to HF [43]. In this study, we observed diminished cardiac function and myocardial fibrosis after MI. However, EA treatment significantly attenuated these pathological processes. Our findings demonstrated that EA treatment markedly increased both the EF and FS, regardless of the treatment duration (either short-term, 7 days, or long-term, 28 days). Additionally, the Sirius Red staining confirmed increased collagen deposition, followed by MI, and EA intervention could reduce the severity of collagen fibrosis in the infarcted hearts.

After MI, the cardiac neural cells can experience ischemic damage, leading to the loss of local autonomic innervation and subsequent neural remodeling. Sympathetic nerve sprouting density in the infarcted area is much higher than that of parasympathetic nerves. This could disrupt the balance of autonomic control and result in a state of heightened sympathetic dominance [44]. Previous research indicated that excessive sympathetic nerve sprouting and subsequent local cardiac neural innervation could lead to exacerbated electrical heterogeneity after initial nerve injury. The enhanced spatial heterogeneity in cardiac sympathetic innervation can amplify the spatial heterogeneity of these electrophysiological characteristics, thereby promoting the occurrence of VAs [6, 45, 46]. HRV reflects the differences in successive cardiac cycles [37], and the dynamic changes in heart rate are influenced by the dual effects of sympathetic and parasympathetic innervations. Our research indicated that after MI, sympathetic activity increased, parasympathetic activity was suppressed, and heightened sensitivity to arrhythmias could be observed. These findings align with our previous research [32], and EA treatment has been shown to mitigate this imbalance, both at 7 days and 28 days after MI.

Excessive sympathetic activation leads to the release of abundant sympathetic neurotransmitters, predominantly NE [47]. NE serves as a powerful biomarker reflecting excessive sympathetic activation and increased risk of mortality in HF patients [48]. In our study, the levels of NE in both serum and heart were significantly higher in the MI group compared to the Sham group. However, increased NE levels could be alleviated in the EA group. These indicated that EA treatment could significantly reduce hyperexcitability in the local and systemic sympathetic nervous system during the repair period of MI. GAP43 is a neuron-specific protein present in axons, which marks neuronal growth through its synthesis in neurons [19].TH is involved in the synthesis of NE and is also considered a marker for the sympathetic nervous system [36, 39, 49]. In this study, we measured the density of GAP43 and TH-positive fibers and their mRNA levels in mice at 28 days after MI. Compared to the Sham group, there was a significant increase in the density of GAP43 and TH-positive fibers, as well as the mRNA levels of GAP43 and TH after MI. This finding is consistent with previous reports [32, 50], and repetitive EA treatments can significantly reduce GAP43 and TH levels. Additionally, it was observed that 7 days after MI, both the protein and mRNA levels of TH were significantly reduced after EA treatment. Consistent with our previous research [32], the present study showed that sympathetic nerve sprouting at the peri-infarct zone in infarcted hearts at 28 days post-MI was more excessive than that in the corresponding zone in normal hearts. EA treatment can ameliorate this abnormality and exhibit inhibitory effects on sympathetic nerve sprouting.

It is well known that inflammation plays a crucial role in MI, as it is closely associated with the repair of necrotic myocardium and directly impacts the prognosis and outcome of MI, as well as being involved in the pathogenesis of remodeling and HF after MI [9, 51, 52]. It is worth noting that macrophages are the predominant immune cell type involved in all phases of MI, and macrophage-derived pro-inflammatory and chemotactic cytokines contribute to the initial hypoxic tissue damage and myocardial remodeling as a function of ischemic injury [53]. Therefore, in this study, we used flow cytometry to detect macrophages in cardiac tissues from different groups of mice after 28 days of MI to detection of inflammatory infiltration in myocardial tissue. We found that EA treatment significantly reduced the number of macrophages in the heart tissue. Surprisingly, we observed no statistical difference in the effect of EA treatment on macrophage polarization at 28 days after MI. Consider the progression of the inflammatory response and the staging of inflammation after MI, as well as the changes in macrophages during its course [54], we dynamically measured macrophage infiltration and macrophage polarization at 1 day, 3 days, and 7 days after MI. We found that EA treatment promoted the polarization of macrophages towards the M2 phenotype and reduced their quantity at 3 days and 7 days after MI. Moreover, it downregulated the expressions of the inflammatory cytokines of TNF-*α*, IL-1*β*, and IL-6 after MI. Therefore, these results suggest that EA treatment can regulate the inflammatory process after MI, promote the polarization of macrophages towards the M2 phenotype, and generate cardioprotective effects.

Previous studies have demonstrated the close association between sympathetic nerve sprouting and the inflammatory process, with macrophages playing a central role in the link between inflammation and sympathetic nerve sprouting after MI [11, 16]. After MI, there is an increased production of NGF due to macrophage infiltration, which is a critical neurotrophic factor promoting active sprouting of sympathetic nerves. It leads to excessive sympathetic innervation. Research has revealed that by promoting macrophage M2 polarization to reduce NGF levels, the excessive sympathetic innervation after MI can be inhibited [17, 18, 20, 55]. However, there is little research on the ways and effects on EA modulate sympathetic nerve remodeling. Therefore, our study investigates whether EA treatment acts through promoting macrophage M2 polarization to decrease NGF levels and subsequently affect sympathetic nerve remodeling. We found that EA treatment significantly reduced the expression of macrophage-related NGF at 7 days after MI, leading to a decrease in upregulated NGF protein levels. Additionally, both protein and mRNA levels of TH were significantly reduced, indicating improvements in sympathetic nerve remodeling. Thus, we propose that EA treatment inhibits NGF expression by promoting macrophage M2 polarization, thereby reducing sympathetic nerve sprouting and improving subsequent sympathetic nerve remodeling, ultimately enhancing cardiac function after MI.

However, this study has limitations. While previous research has confirmed that macrophage polarization can affect NGF secretion and, consequently, impact sympathetic nerve remodeling, it is important to acknowledge that acupuncture has multiple mechanisms of action. Thus, we have not completely ruled out other pathways that may contribute to these effects, and further validation through macrophage ablation would be necessary. Moreover, it is also important to investigate whether the central sympathetic nervous system is involved in this process.

## 5. Conclusions

In conclusion, our study suggests that EA treatment can reduce sympathetic nerve remodeling and improve cardiac function after MI. This could be achieved by accelerating the inflammatory response, promoting macrophage M2 polarization, and subsequently reducing NGF levels. EA treatment may serve as a complementary therapy to promote myocardial protection after MI.

## Figures and Tables

**Table 1:** Primer sequences for RT-qPCR analysis.

Gene name	Primer sequences 5′→3′
TNF-*α*	Forward: TCTTCTCATTCCTGCTTGTGG Reverse: GGTCTGGGCCATAGAACTGA

IL-1*β*	Forward: AGAAGCTGTGGCAGCTACCTG Reverse: GGAAAAGAAGGTGCTCATGTCC

IL-6	Forward: GCTACCAAACTGGATATAATCAGGA Reverse: CCAGGTAGCTATGGTACTCCAGAA

TH	Forward: GTCACGTCCCCAAGGTTCAT Reverse: CCTCGAATACCACAGCCTCC

GAP43	Forward: ACCTAAGGAAAGTGCCCGAC Reverse: GAGAGACAGGGTTCAGGTGG

GAPDH	Forward: AAATGGTGAAGGTCGGTGTGAAC Reverse: CAACAATCTCCACTTTGCCACTG

## Data Availability

The datasets used and/or analyzed during the current study are available from the corresponding author upon reasonable request.

## Abbreviations

EA: Electroacupuncture
PC6: Neiguan acupoint
MI: Myocardial infarction
LAD: Left anterior descending
LVEDD: Left ventricular end-diastolic diameter
LVESD: Left ventricular end-systolic diameter
EF: Ejection fraction
FS: Fractional shortening
HR: Heartbeats per minute
HRV: Heart rate variability analysis
NE: Norepinephrine
GAP43: Growth-associated protein 43
TH: Tyrosine hydroxylase
IL-1*β*: Interleukin-1*β*
IL-6: Interleukin-6
TNF-*α*: Tumor necrosis factor-*α*
NGF: Nerve growth factor.
